# Supplementary material for: Induced responses contribute to rapid adaptation of Spirodela polyrhiza to herbivory by Lymnaea stagnalis
Source: Commun Biol. 2024 Jan 11;7:81. doi: 10.1038/s42003-023-05706-0 (PMC10781955; doi:10.1038/s42003-023-05706-0)
Supplement: Supplementary file 2 — Supplementary material [file 42003_2023_5706_MOESM2_ESM.pdf]

Supplementary Material

Induced responses contribute to rapid plant adaptation to herbivory

Antonino Malacrinò, Laura Böttner, Sara Nouere, Meret Huber, Martin Schäfer, Shuqing Xu

Contents

Supplementary figures **2**

Supplementary tables **6**

Supplementary methods - water nutrients analysis **9**

Supplementary methods - metabolomics **10**

Supplementary figures

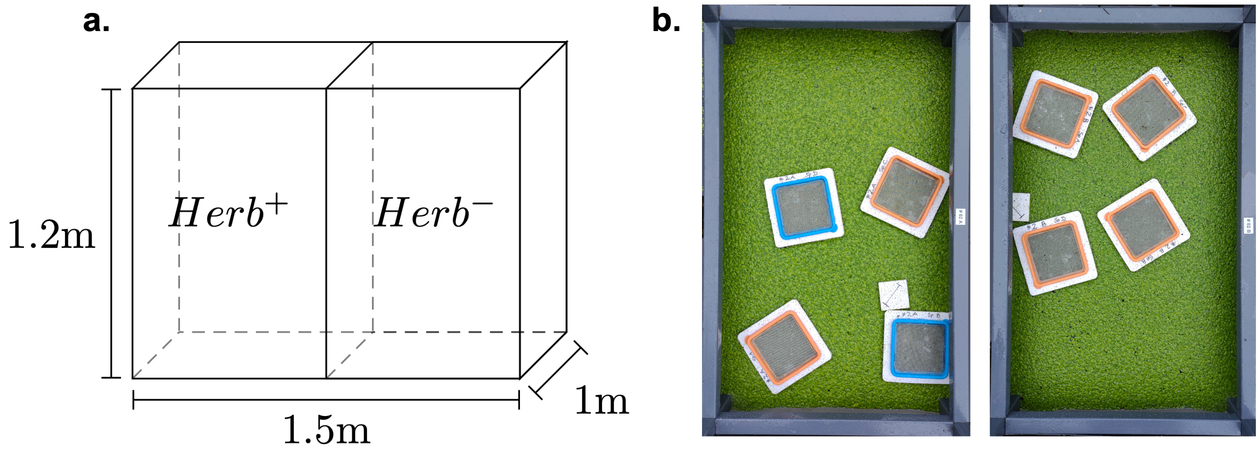

Figure S1. (a) Schematic representation of each pond. (b) Example of control (left) and herbivory (right) cages showing the floating boxes.

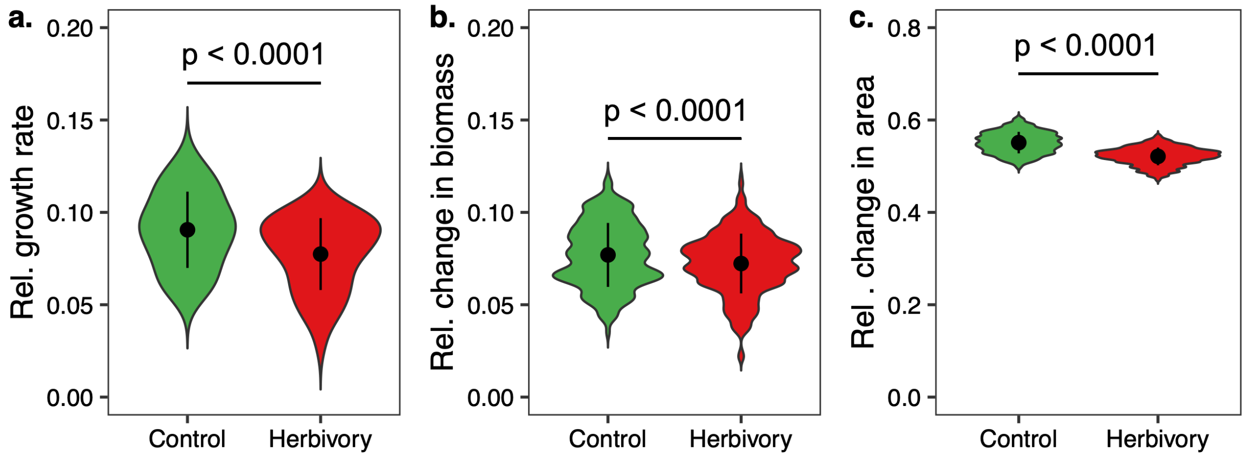

Figure S2. Relative growth rate (**a**; F = 36.72; df = 1; $p < 0.0001$), and change in biomass (**b**; F = 972.74; df = 1; $p < 0.0001$) and surface area (**c**; F = 28132; df = 1; $p < 0.0001$) within 2 weeks for plants exposed to 8 weeks of multigenerational herbivory or control conditions. Values for plots (**b**) and (**c**) are calculated by permutation ($n = 999$) with a set of reference fronds collected before the bioassay. For each group, dots represent mean values and bars show the standard deviation.

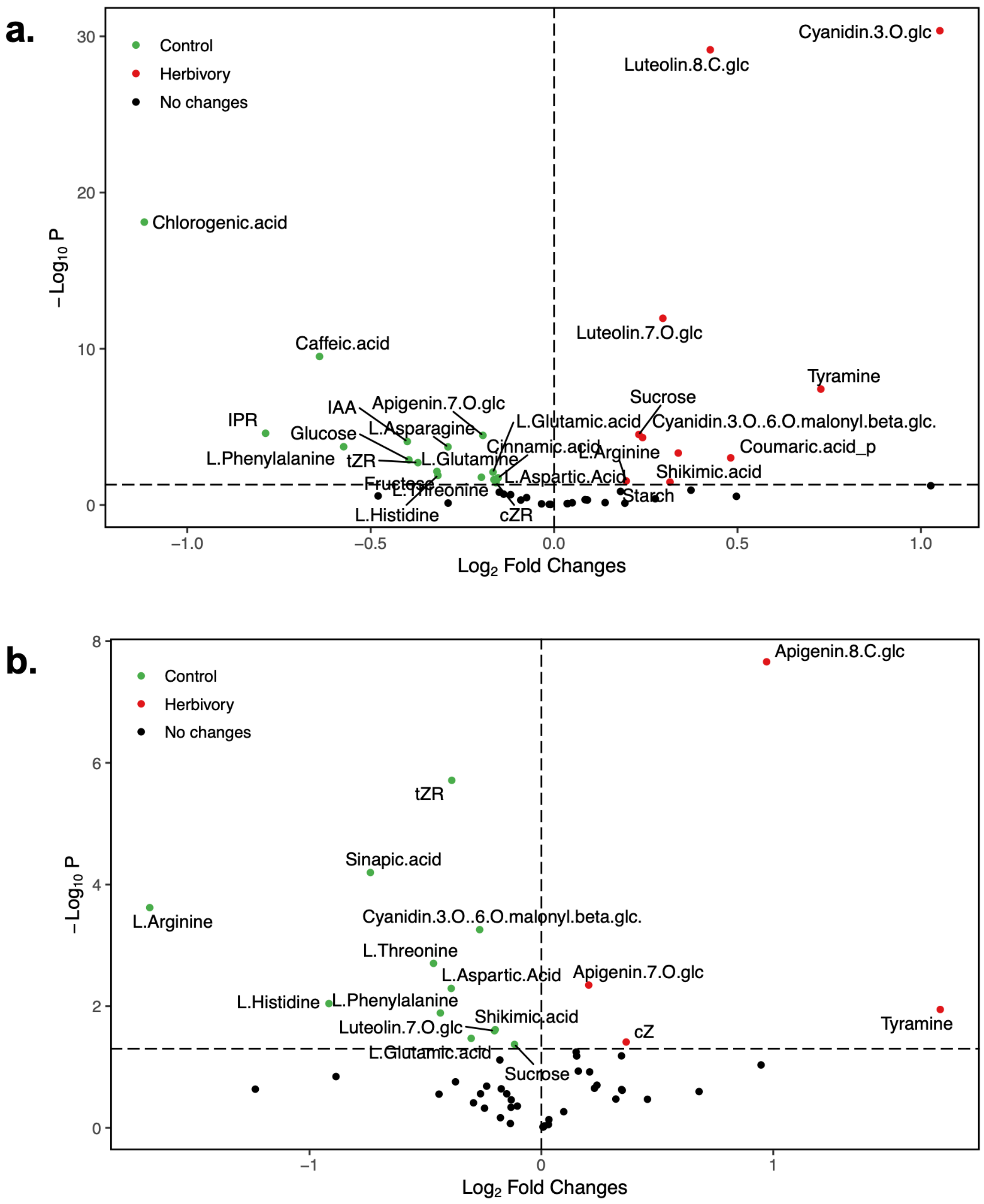

Figure S3. Volcano plot showing the metabolites of mixed popualtions with higher abundance in either control (left) or herbivory (right) treatments for first (**a**; week 12) and second (**b**; week 58) growing season.

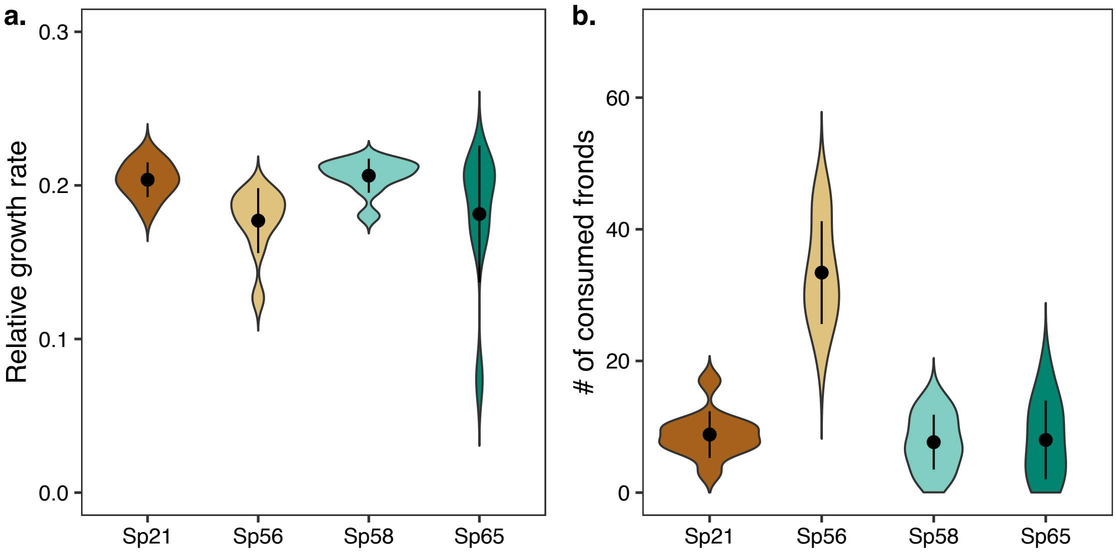

Figure S4. Relative intrinsic growth rate of each Spirodela polyrhiza genotype grown separatly under sterile and controlled conditions indoors (**a**; $F_{3,\,35} = 3.51$, $p = 0.02$), and number of consumed fronds for each of them after being exposed to 6 h of herbivory by a single snail (**b**; $F_{3,\,44} = 58.62$, $p < 0.0001$). For each group, dots represent mean values and bars show the standard deviation. **Methods**: To compare intrinsic growth rates and resistance of our four *Spirodela polyrhiza* genotypes, we grew gnotobiotic fronds of all genotypes separately in controlled conditions at 23 °C. Gnotobiotic plants were pre-cultivated under sterile conditions in full N-Medium for four weeks, before being transferred for pre-adaptation to 1:10 N-Medium under sterile conditions for another week. We then started the bioassays adding 30 gnotobiotic plants, separately for each genotype (n=10 boxes for Sp21, Sp56, Sp58, n=8 boxes of Sp65), to an autoclaved plastic box with a fine metal net on top of the lid to allow gas exchange and filled with 800 mL of fresh sterile 1:10 N-Medium. First, the 30 starting fronds were allowed to propagate for 13 days before harvested, and resulting fronds were counted. Second, we transferred 50 fronds from each box to a plastic filled with 200 mL of 1:10 N-Medium, we added one snail (from our laboratory culture, starved in 1:10 N-Medium for 24 prior) to each cup. Snails were removed after 6h, and we counted the number of consumed fronds.

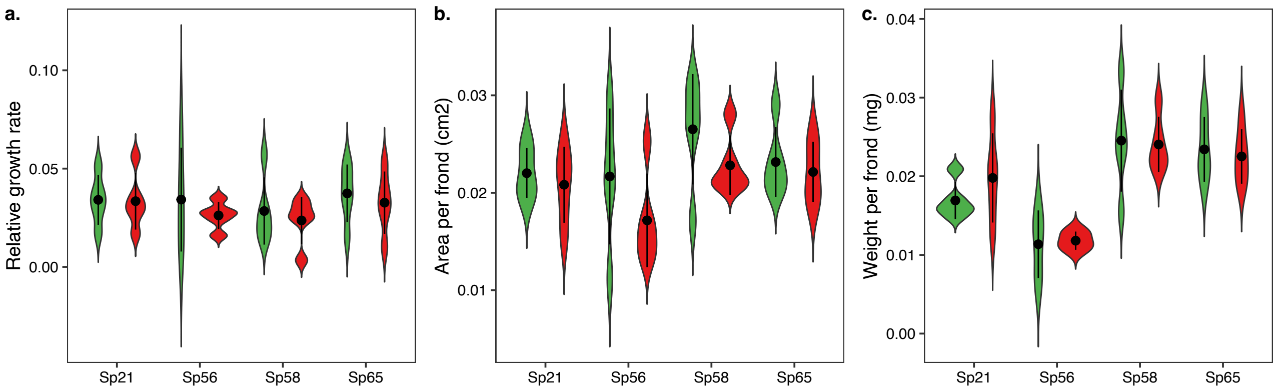

Figure S5. Relative growth rate (**a**), area per frond (**b**), and dry weight per frond (**c**) after a 13-days growth assay of individual *Spirodela polyrhiza* genotypes grown for 8 weeks in presence (red) or absence (green) of herbivores in outdoor ponds. Results from data analysis are reported in Tab. S5 below.

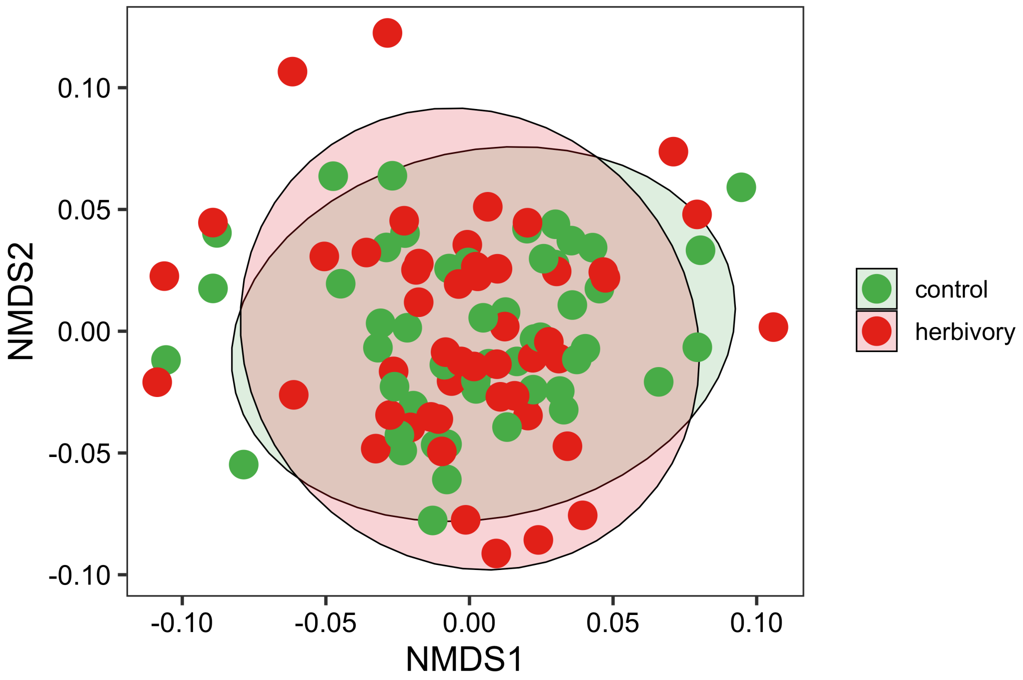

Figure S6. NMDS showing the overlap between water nutrient content in control and herbivory cages in outdoor ponds (data collected at weeks 4, 6, 8, 10, and 12). PERMANOVA analysis confirmed no differences between the two treatments ($F_{1,\,98} = 0.38$, $p = 0.41$; 999 permutations stratified over "pond" and "sampling timepoint").

Supplementary tables

Table S1. Geographical origins and Unified ID for each duckweed genotype used in our study.

Genotype ID	Geographical origin	Unified ID
Sp21	Porstendorf, Germany	9500
Sp56	Pukkila, Finland	9256
Sp58	Szarvas, Hungary	9560
Sp65	Delta del Po, Veneto, Italy	9413

Table S2. Linear mixed-effect models testing the influence of length of time of experimental evolution and treatment (herbivory/control) on *Spirodela polyrhiza* frond area and biomass.

		Area per frond		Weight per frond	
**Factor**	**df**	**F**	**p**	**F**	**p**
Timepoint	1	8.05	0.0052	2.51	0.1158
Treatment	1	103.19	<0.0001	7.10	0.0086
Timepoint × Treatment	1	0.39	0.5324	0.61	0.4338

Table S3. Linear mixed-effect model testing the effect of treatment (herbivory/control) and genotype (Sp21, Sp56, Sp58, and Sp65) on the relative frequency of each *Spirodela polyrhiza* genotype within the experimental ponds after 12 weeks of experimental evolution approach in outdoor ponds. Both the pond ID and the sampling week were used as random factors.

Factor	df	F	p
Treatment	1	2.08	0.15
Genotype	3	52.25	<0.0001
Treatment × Genotype	3	9.23	<0.0001

Table S4. Linear mixed-effect models testing the influence of genotype and treatment (herbivory/control) on *Spirodela polyrhiza* frond area and biomass on individual genotypes grown in greenhouse for 8 weeks under herbivory or control conditions.

		Relative growth rate		Area per frond		Weight per frond	
**Factor**	**df**	**F**	**p**	**F**	**p**	**F**	**p**
Genotype	3	0.87	0.46	2.63	0.07	22.81	<0.0001
Treatment	1	1.11	0.30	3.68	0.06	0.16	0.69
Timepoint × Treatment	3	0.12	0.94	1.42	0.73	0.49	0.68

Table S5. PERMANOVA testing the effect of treatment (herbivory/control) and length of experimental evolution (week) on the structure of bacterial, fungal, and algal communities associated with *Spirodela polyrhiza.*

		Bacteria and fungi			Algae		
**Factor**	**df**	R^2	**F**	**p**	R^2	**F**	**p**
Treatment	1	0.05	2.54	0.007	0.06	4.41	0.036
Week	1	0.15	7.16	0.001	0.34	22.54	0.001
Treatment × Week	1	0.02	1.04	0.186	0.04	3.13	0.070

Table S6. Linear mixed-effect models testing the effect of genotype (Sp21, Sp56, Sp58, and Sp65) and treatment (herbivory/control) on the growth (changes in biomass and area) and resistance (consumed biomass and area) of single genotype of *Spirodela polyrhiza* grown in the experimental ponds.

		Change in biomass		Change in area		Consumed biomass		Consumed area	
**Factor**	**df**	**F**	**p**	**F**	**p**	**F**	**p**	**F**	**p**
Genotype	3	2.83	0.04	0.11	0.95	137.94	<0.001	24.25	<0.001
Treatment	1	0.26	0.61	0.37	0.54	41.95	<0.001	4.20	0.04
Genotype × Treatment	3	0.23	0.87	0.34	0.79	78.71	<0.001	32.57	<0.001

Table S7. Linear mixed-effect model testing the effect of treatment (herbivory/control) on the number of consumed fronds of single genotypes after 24h of herbivory for each *Spirodela polyrhiza* genotype, grown for 8 weeks outdoors in cages with or without herbivores, but protected from snails using fine nets.

Genotype	F	df	p
Sp21	0.001	1	0.96
Sp56	0.037	1	0.84
Sp58	2.458	1	0.11
Sp65	3.669	1	0.05

Table S8. Taxa differentially abundant between plants (genotypes Sp21 and Sp65) grown under control or herbivory-induced microbiota environment.

Genotype	Marker	Treatment with higher abundance	Genus	Number of ASVs
Sp21	16S	Herbivory	*Cetobacterium*	3
		Control	-	-
Sp65	16S	Herbivory	*Bacteroides*	1
		Control	*Ralstonia*	1
			Unid. Muribaculaceae	3
			Clostridium	1
			Unid. Lachnospiraceae	1
			Cetobacterium	2

Table S9. Linear-mixed effects models testing the influence of herbivory on water nutrient levels (and pH) during the first growing season. Both the pond ID and the sampling timepoints were used as random factors.

Nutrient	Control (mean ± SD)	Herbivore (mean ± SD)	df	F	p
Cl	25.90 ± 4.44	25.27 ± 4.96	1	4.77	0.03
NO_2	4.68 ± 1.24	4.63 ± 1.12	1	0.19	0.66
NO_3	8.69 ± 5.50	8.28 ± 5.21	1	2.66	0.10
PO_4	1.67 ± 1.16	1.73 ± 1.06	1	0.25	0.61
SO_4	11.98 ± 4.73	11.58 ± 4.66	1	4.42	0.03
Na	25.48 ± 3.21	24.98 ± 3.73	1	2.22	0.14
NH_4	1.95 ± 2.84	1.91 ± 2.76	1	0.35	0.55
K	35.37 ± 7.86	34.72 ± 8.63	1	0.67	0.41
Ca	33.44 ± 6.30	32.87 ± 6.35	1	0.81	0.37
Mg	4.44 ± 0.61	4.37 ± 0.68	1	1.37	0.24
pH	7.49 ± 0.48	7.50 ± 0.48	1	0.36	0.54

Supplementary methods - water nutrients analysis

While our treatment cages shared the same water body of a pond, thus per se allowing exchange of nutrients, we tested individual nutrient levels across treatments and ponds using ion chromatography (792 basic IC, 732 detector, Metrohm, Herisau, Switzerland). Samples were taken every two weeks (from experimental setup to week 12) during the first season (n=10 per treatment cage) and once during the second growth season at week 58 (n=7 per treatment cage). We sampled 10 mL per cage for anion and cation analysis, respectively. When collecting samples from each cage, we pooled water from five different locations at a depth of 10 cm below the water surface. Anion samples were measured right away, while pH of cation samples was adjusted to pH 3 using 25 % HNO_3 before storage at -20°C until analysis. Anions (Cl, NO_2, NO_3, PO_4, SO_4) were measured using: column Metrosep A Supp 5 150, 4.0 x 150 mm, eluent 3.2 mM Na_2CO_3 and 1 mM $NaHCO_3$, flow rate 0.7 mL/min, injection volume 20 µl. Cations (Na, NH4, K, Ca, Na) were measured using: column Metrosep C2 250, 4.0 x 250 mm, eluent 1.7 mmol/L nitric acid, 0.7 mmol/L dipicolinic acid, flow rate 0.9 mL/min, injection volume 20 µl. Quantification was based on external standard ion mixture using a 6-point-reference curve (0.5, 5, 10, 25, 50, 100 mg/L for anions and 0.5, 5, 10, 15, 25, 50 mg/L for cations). Additionally, throughout the growth seasons we measured the pH of each pond using a Cond3310, (WTW, Xylem Analytics) instrument.

The effect of herbivory on water nutrients and pH was tested by fitting a linear mixed effects model specifying treatment (herbivory/control) as fixed factor, and pond and timepoint as random effects, using the formula: $\sim$ treatment * (1|timepoint) * (1|pond). In addition, we tested the effects of overall nutrient content through PERMANOVA on data collected at week 4, 6, 8, 10, and 12 (999 permutations, stratified over "pond" and "sampling timepoint").

Supplementary methods - metabolomics

Extraction and analysis of diverse primary and secondary metabolites was done similar as described by Schäfer et al. [1]. A detailed list of the analyzed compound and the respective analytical methods can be found in Table S10-16. In brief, approximately 10 mg fine ground, freeze dried plant tissue was extracted with acidified methanol containing the following internal standards: 10 ng D_6-ABA (OlChemIm); 10 ng D_5-JA (Sigma-Aldrich); 10 ng D_4-SA (OlChemIm); 0.5 ng D_5-tZ (OlChemIm); 0.1 ng D_6-IP (OlChemIm); 0.1 ng D_6-IPR (OlChemIm); 1 ng $^{13}C_6$-IAA (OlChemIm); 3 ng 4-methylumbelliferone (4-MU) (Sigma-Aldrich). For the analysis of different high abundant compounds, a small aliquot (A1) of the extract was taken aside, while the rest of the extract (E1) was used for further processing. High abundant compounds, such as amino acids and related compounds were analyzed in a 1:100 dilution of A1 in an aqueous mix of isotope-labeled amino acids (algal amino acid mixture-^{13}C-^{15}N; Sigma-Aldrich; Method 1A and 1B). Soluble sugars were measured in a 1:125 dilution of A1 in 70% methanol containing sorbitol as internal standard (Fluka Biochemika; Method 1C). The major secondary metabolites were analyzed in the rest of the aliquot (A1) without further dilution (Method 1D). After a second extraction of the plant tissue with acidified methanol this supernatant was combined with the first extract (E1) and subsequently purified on a reversed phase (RP) solid phase extraction (SPE) column (HR-X, Macherey-Nagel) and a mixed-mode RP-cation exchange SPE column (HR-XC, Macherey-Nagel). The compounds were bound to the HR-XC column, different washing steps were applied (e.g. to remove CK-phosphates) and the analytes of interest were stepwise eluted from the column to generate a fraction containing the acidic phytohormones, such as JA, ABA and IAA, as well as a fraction containing the CKs. The eluted fractions were analyzed directly (Method 2A) or after an additional concentration step (Method 2B) and (Method 3) dependent on the abundance and instrument sensitivity for the respective compounds. Starch content was determined from the remaining material (pellet) after the two rounds of extraction with acidified methanol. For this, the material was subjected to two additional washing steps with 50% ethanol at 80 °C to remove soluble sugars. Subsequently, a sub-fraction of the material was resuspended in water and incubated at 98 °C to gelatinize the starch, which was then digested to glucose by amyloglucosidase (from Aspergillus niger, Roche) and ¸ -amylase (from porcine pancreas, Sigma) at 37 °C. The glucose content was then measured in a 1:250 dilution of this solution in 70% methanol containing sorbitol as internal standard the same way as for the soluble sugars before (Method 1C).

The major secondary metabolites (Method 1D) were analyzed by HPLC-PDA. Chromatographic separation was done on a Shimadzu Nexera XR LC-System equipped with an EC 4/3 Nucleodur Sphinx RP pre-column (5 µm, Macherey-Nagel) and a Nucleodur Sphinx RP column (250x4.6 mm, 5µm, Macherey-Nagel). The mobile phase comprised 0.2% formic acid (Fisher Chemical), 0.1% acetonitrile (Fisher Chemical) in water as Solvent A and acetonitrile (Fisher Chemical) as Solvent B. The column oven was set to 20 °C and the flow rate to 1300 µL/min. Measurement was performed with a PDA detector.

Other compounds (Method 1A, 1B, 1C, 2A, 2B and 3) were analyzed by LC-MS/MS. Chromatographic separation for was done on a Shimadzu Nexera X3 LC-System. For method 1A, 1B, 2A, 2B and 3 the system was equipped with an Agilent 1290 infinity II inline filter (0,3 µm) and a ZORBAX RRHD Eclipse XDB-C18 column (3x50 mm, 1.8 µm; Agilent Technologies). The mobile phase comprised 0.05% formic acid (Fisher Chemical), 0.1% acetonitrile (Fisher Chemical) in water as Solvent A and methanol (Fisher Chemical) as Solvent B. The column oven was set to 42 °C and the flow rate to 500 µL/min. For method 1C the system was equipped with an Agilent 1290 infinity II inline filter (0,3 µm) and an apHera NH2 column (150x4.6 mm, 5 µm; Supelco). The mobile phase comprised 0.1% acetonitrile (Fisher Chemical) in water as Solvent A and acetonitrile (Fisher Chemical) as Solvent B. The column oven was set to 25 °C and the flow rate to 700 µL/min. The measurements were performed on a Shimadzu LCMS-8060 equipped with an ESI source, which was operated in multi-reaction-monitoring (MRM) modus. Settings were as follows: Nebulizing Gas Flow: 3 L/min; Heating Gas Flow: 10 L/min; Drying Gas Flow: 10 L/min; Interface Temperature: 300 °C; DL Temperature

250 °C; Heat Block Temperature: 400 °C; CID Gas: 270 kPa; Q1 Resolution: Unit; Q3 Resolution: Unit.

The gradient programs for all methods are given in Table S17-23 and the detector settings (MRM-settings or absorption wavelength, respectively) in Table S10-16.

Table S10. MRM-settings and retention times of analytes of Method 1A.

**Analyte**	**RT [min]**	**Q1 [m/z]**	**Q3 [m/z]**	**Dwell time [ms]**	**CE [V]**	**Q1/Q3 Pre Bias [V]**	**ISTD** [a]
Ala	0.450	(+)90.05	44.20	20	-13	-10/-18	$^{13}C_3$,$^{15}N_1$-Ala
Arg	0.420	(+)175.12	60.20	20	-14	-20/-24	$^{13}C_6$,$^{15}N_4$-Arg
Asp	0.450	(+)134.04	88.20	20	-12	-13/-16	$^{13}C_4$,$^{15}N_n$-Asx_{Asp} [b]
Glu	0.450	(+)148.06	102.15	20	-13	-10/-19	$^{13}C_5$,$^{15}N_n$-Glx_{Glu} [b]
Pro	0.485	(+)116.07	70.20	20	-17	-11/-20	$^{13}C_5$,$^{15}N_1$-Pro
Ser	0.435	(+)106.05	60.20	20	-14	-10/-23	$^{13}C_3$,$^{15}N_1$-Ser
Thr	0.450	(+)120.07	74.20	20	-12	-12/-30	$^{13}C_4$,$^{15}N_1$-Thr
Asn	0.435	(+)133.06	87.20	20	-11	-13/-16	$^{13}C_4$,$^{15}N_n$-Asx_{Asn} [b]
Gln	0.450	(+)147.08	130.15	20	-15	-10/-13	$^{13}C_5$,$^{15}N_n$-Glx_{Gln} [b]
Val	0.627	(+)118.09	72.20	20	-12	-22/-28	$^{13}C_5$,$^{15}N_1$-Val
Ile	1.165	(+)132.10	86.15	122	-11	-20/-20	$^{13}C_6$,$^{15}N_1$-Ile
Leu	1.255	(+)132.10	86.15	122	-11	-20/-20	$^{13}C_6$,$^{15}N_1$-Leu
Phe	2.520	(+)166.09	120.20	247	-15	-20/-20	$^{13}C_9$,$^{15}N_1$-Phe
Trp	3.260	(+)205.10	188.10	32	-10	-20/-20	$^{13}C_9$,$^{15}N_1$-Phe (1.76)
		(+)205.10	118.20	32	-26	-20/-20	
$^{13}C_3$, $^{15}N_1$-Ala	0.450	(+)94.06	47.20	20	-13	-10/-18	
$^{13}C_6$, $^{15}N_4$-Arg	0.438	(+)185.13	64.15	20	-14	-20/-24	
$^{13}C_4$, $^{15}N_n$-Asx	0.450	(+)139.06	92.25	20	-12	-13/-16	
$^{13}C_5$, $^{15}N_n$-Glx	0.450	(+)154.07	107.20	20	-13	-10/-19	
$^{13}C_5$, $^{15}N_1$-Pro	0.485	(+)122.08	75.20	20	-17	-11/-20	
$^{13}C_3$, $^{15}N_1$-Ser	0.435	(+)110.06	63.20	20	-14	-10/-23	
$^{13}C_4$, $^{15}N_1$-Thr	0.450	(+)125.08	78.20	20	-12	-12/-30	
$^{13}C_5$, $^{15}N_1$-Val	0.627	(+)124.10	77.20	20	-12	-22/-28	
$^{13}C_6$, $^{15}N_1$-Ile	1.165	(+)139.12	92.25	122	-11	-20/-20	
$^{13}C_6$, $^{15}N_1$-Leu	1.255	(+)139.12	92.25	122	-11	-20/-20	
$^{13}C_9$, $^{15}N_1$-Phe	2.520	(+)176.11	129.25	247	-15	-20/-20	

RT: retention time
CE: collision energy
ISTD: internal standard
Qualifiers are highlighted in grey
[a] Incl. matrix corrected response factor ($n_{Analyte}$ = x * n_{ISTD}) in brackets
[b] Asx and Glx handled as Asn, Asp, Glu or Gln equivalents, respectively

Table S11. MRM-settings and retention times of analytes of Method 1B.

Analyte	RT [min]	Q1 [m/z]	Q3 [m/z]	Dwell time [ms]	CE [V]	Q1/Q3 Pre Bias [V]	ISTD[a]
Gly	0.46	(+)76.04	30.20	50	-12	-14/-11	$^{13}C_2$,$^{15}N_1$-Gly
His	0.44	(+)156.08	110.20	50	-15	-30/-21	$^{13}C_6$,$^{15}N_3$-His
Met	0.75	(+)150.06	61.15	50	-22	-10/-24	$^{13}C_5$,$^{15}N_1$-Met
Shikimic acid	0.63	(-)173.05	93.10	23	15	12/21	$^{13}C_9$,$^{15}N_1$-Tyr (37.77)
		(-)173.05	111.20	23	13	12/10	
Tyr	1.13	(+)182.08	136.20	50	-15	-18/-14	$^{13}C_9$,$^{15}N_1$-Tyr
Tyramine	1.16	(+)138.09	121.15	23	-15	-20/-20	$^{13}C_9$,$^{15}N_1$-Tyr (0.51)
		(+)138.09	77.15	23	-30	-20/-20	
Tryptamine	3.28	(+)161.15	144.15	297	-15	-20/-20	$^{13}C_9$,$^{15}N_1$-Tyr (0.36)
		(+)161.15	117.15	297	-25	-20/-22	
Apigenin	4.12	(-)269.05	151.20	147	25	30/14	$^{13}C_9$,$^{15}N_1$-Tyr (0.96)
		(-)269.05	149.15	147	24	29/28	
Luteolin	4.025	(-)285.04	132.20	147	48	30/26	$^{13}C_9$,$^{15}N_1$-Tyr (3.44)
		(-)285.04	175.20	147	26	19/11	
$^{13}C_2$, $^{15}N_1$-Gly	0.46	(+)79.04	32.10	50	-12	-14/-11	
$^{13}C_6$, $^{15}N_3$-His	0.44	(+)165.09	118.20	50	-15	-30/-21	
$^{13}C_5$, $^{15}N_1$-Met	0.75	(+)156.07	63.15	50	-22	-10/-24	
$^{13}C_9$, $^{15}N_1$-Tyr	1.13	(+)192.11	145.20	50	-15	-18/-14	

RT: retention time
CE: collision energy
ISTD: internal standard
Qualifiers are highlighted in grey
[a] Incl. matrix corrected response factor ($n_{Analyte} = x * n_{ISTD}$) in brackets

Table S12. MRM-settings and retention times of analytes of Method 1C.

Analyte	RT [min]	Q1 [m/z]	Q3 [m/z]	Dwell time [ms]	CE [V]	Q1/Q3 Pre Bias [V]	ISTD[a]
Fructose	7.8	(-)179.06	88.90	19	9	12/13	Sorbitol[b]
		(-)179.06	59.00	19	17	12/11	
		(-)179.06	71.00	19	16	11/11	
Glucose	9.2	(-)179.06	88.90	19	9	12/13	Sorbitol[b]
		(-)179.06	59.00	19	17	12/11	
		(-)179.06	71.00	19	16	11/11	
Sucrose	8.4	(-)341.11	89.10	19	23	12/10	Sorbitol[b]
		(-)341.11	179.15	19	15	30/30	
		(-)341.11	59.05	19	36	29/23	
Sorbitol	10.8	(-)181.07	88.85	19	16	11/17	
		(-)181.07	59.00	19	22	11/10	
		(-)181.07	71.00	19	22	30/29	

RT: retention time
CE: collision energy
ISTD: internal standard
Qualifiers are highlighted in grey
[a] Incl. matrix corrected response factor ($n_{Analyte} = x * n_{ISTD}$) in brackets
[b] Response factor calculated based on an external dilution curve run with each batch of samples

Table S13. Absorption and retention times of analytes of Method 1D. Quantification based on an external dilution curve with identical standards, except for the putative chlorogenic acid isomere that was based on the chlorogenic acid standard, and cyanidin-3-O-(6-O-malonyl-beta-glucoside) that was quantified based on the molar quantity of the cyanidin-3-O-glucoside standard.

Analyte	RT [min]	Absortion wave lenght [nm]
Cyanidin-3-O-glucoside	7.055	517
Chlorogenic acid	8.055	328
Putative Chlorogenic acid isomere	8.210	328
Cyanidin-3-O-(6-O-malonyl-beta-glucoside)	9.410	517
Luteolin-8-C glucoside	11.295	348
Apigenin-8-C-glucoside	12.495	337
Luteolin-7-O-glucoside	13.085	348
Apigenin-7-O-glucoside	14.325	337

RT: retention time

Table S14. MRM-settings and retention times of analytes of Method 2A

Analyte	RT [min]	Q1 [m/z]	Q3 [m/z]	Dwell time [ms]	CE [V]	Q1/Q3 Pre Bias [V]	ISTD
SA	2.57	(-)137.02	92.95	20	19	27 / 19	D_4-SA
		(-)137.02	65.00	20	32	27 / 10	
ABA	2.71	(-)263.13	153.10	20	13	18 / 29	D_6-ABA
		(-)263.13	204.15	20	20	18 / 12	
JA	3.20	(-)209.12	59.00	20	14	14 / 11	D_5-JA
		(-)209.12	40.95	20	44	14 / 14	
JA-Ile	4.57	(-)322.20	130.10	135	22	24 / 20	D_5-JA (0.084)
		(-)322.20	128.15	135	22	24 / 26	
OPDA	6.21	(-)291.20	165.25	135	22	20 / 10	D_5-JA^b
		(-)291.20	247.15	135	20	20 / 23	
D_4-SA	2.56	(-)141.05	97.00	20	19	27 / 15	
		(-)141.05	69.05	20	32	27 / 17	
D_6-ABA	2.70	(-)269.17	159.20	20	13	18 / 30	
		(-)269.17	207.20	20	20	18 / 20	
D_5-JA	3.19	(-)214.15	62.00	20	14	14/ 10	
		(-)214.15	42.00	20	44	14 / 15	

RT: retention time
CE: collision energy
ISTD: internal standard
Qualifiers are highlighted in grey
[a] Incl. matrix corrected response factor ($n_{Analyte} = x * n_{ISTD}$) in brackets
[b] Relative quantification

Table S15.MRM-settings and retention times of analytes of Method 2B.

Analyte	RT [min]	Q1 [m/z]	Q3 [m/z]	Dwell time [ms]	CE [V]	Q1/Q3 Pre Bias [V]	ISTD[a]
Caffeic acid	2.275	(-) 179.03	135.05	20	18	12 / 26	4-MU[b]
		(-) 179.03	134.15	20	25	12 / 12	
		(-) 179.03	107.10	20	24	19 / 21	
p-Coumaric acid	2.624	(-) 163.04	119.10	20	17	11 / 11	4-MU[b]
		(-) 163.04	93.05	20	32	11 / 19	
Sinapic acid	2.76	(+)225.08	207.15	20	-9	-15 / -23	4-MUb
		(+)225.08	119.20	20	-19	-15 / -12	
IAA	3.237	(+)176.07	130.10	20	-16	-12 / -23	$^{13}C_6$-IAA (0.42)
		(+)176.07	103.20	20	-31	-12 / -10	
Cinamic acid	5.002	(-) 147.05	103.10	100	15	17 / 20	4-MU[b]
		(-) 147.05	77.10	100	23	10 / 18	
$^{13}C_6$-IAA	3.237	(+)182.09	109.20	20	-31	-12 / -19	
		(+)182.09	136.25	20	-16	-12 / -13	
4-MU	3.344	(+)177.05	77.20	20	-35	-12 / -30	
		(+)177.05	105.20	20	-21	-12 / -20	

RT: retention time
CE: collision energy
ISTD: internal standard
Qualifiers are highlighted in grey
[a] Incl. matrix corrected response factor ($n_{Analyte} = x * n_{ISTD}$) in brackets
[b] Relative quantification

Table S16. MRM-settings and retention times of analytes of Method 3.

Analyte	RT [min]	Q1 [m/z]	Q3 [m/z]	Dwell time [ms]	CE [V]	Q1/Q3 Pre Bias [V]	ISTD[a]
tZ	2.527	(+)220.12	136.10	47	-20	-20 / -20	D_5-tZ
		(+)220.12	119.15	47	-33	-22 / -12	
cZ	2.83	(+)220.12	136.10	47	-20	-20 / -20	D_5-tZ (0.94)
		(+)220.12	119.15	47	-33	-22 / -12	
tZR	4.292	(+)352.16	220.15	30	-20	-20 / -20	D_5-tZ (0.17)
		(+)352.16	136.15	30	-30	-20 / -20	
cZR	4.696	(+)352.16	220.15	30	-20	-20 / -20	D_5-tZ (0.17)
		(+)352.16	136.15	30	-33	-20 / -20	
IP	5.648	(+)204.12	136.10	30	-15	-20 / -20	D_6-IP
		(+)204.12	119.10	30	-30	-20 / -20	
IPR	6.39	(+)336.17	204.20	47	-20	-20 / -20	D_6-IPR
		(+)336.17	136.10	47	-30	-20 / -20	
D_5-tZ	2.52	(+)225.15	137.20	47	-20	-21 / -22	
		(+)225.15	119.20	47	-36	-15 / -12	
D_6-IP	5.511	(+)210.16	137.20	30	-15	-20 / -20	
		(+)210.16	119.15	30	-33	-14 / -22	
D_6-IPR	6.363	(+)342.20	210.25	47	-20	-20 / -20	
		(+)342.20	137.15	47	-30	-20 / -20	

RT: retention time
CE: collision energy
ISTD: internal standard
Qualifiers are highlighted in grey
[a] Incl. matrix corrected response factor ($n_{Analyte} = x * n_{ISTD}$) in brackets

Table S17. Solvent settings used for Method 1A.

Time [min]	B [%]
0.0	2
1.5	2
3.5	100
4.5	100
5.0	2
6.0	2

Table S18. Solvent settings used for Method 1B.

Time [min]	B [%]
0.0	2
1.5	2
3.5	100
4.5	100
5.0	2
6.0	2

Table S19. Solvent settings used for Method 1C.

Time [min]	B [%]
0	80
13	60
14	80
20	80

Table S20. Solvent settings used for Method 1D.

Time [min]	B [%]
0.0	10
8.0	21
18.0	49
18.1	100
19.0	100
19.1	10
24.0	10

Table S21. Solvent settings used for Method 2A.

Time [min]	B [%]
0.0	10
0.5	10
1.0	55
4.5	65
5.5	100
6.5	100
7.0	10
8.0	10

Table S22. Solvent settings used for Method 2B.

Time [min]	B [%]
0.0	10
0.5	10
1.0	39
3.5	41
3.6	50
8.0	60
8.5	100
9.5	100
10.0	10
11.0	10

Table S23. Solvent settings used for Method 3.

Time [min]	B [%]
0.0	5
0.5	5
0.7	15
3.5	25
6.5	70
6.7	100
7.7	100
8.0	5
9.0	5

References

[1] M. Schäfer, C. Brütting, I. T. Baldwin, M. Kallenbach, High-throughput quantification of more than 100 primary-and secondary-metabolites, and phytohormones by a single solid-phase extraction based sample preparation with analysis by UHPLC– HESI–MS/MS. Plant Methods, 12, 1-18 (2016).
